# Beneficial Effects of Ursodeoxycholic Acid on Metabolic Parameters and Oxidative Stress in Patients with Type 2 Diabetes Mellitus: A Randomized Double-Blind, Placebo-Controlled Clinical Study

**DOI:** 10.1155/2024/4187796

**Published:** 2024-02-29

**Authors:** Biljana Lakić, Ranko Škrbić, Snežana Uletilović, Nebojša Mandić-Kovačević, Milkica Grabež, Mirna Popović Šarić, Miloš P. Stojiljković, Ivan Soldatović, Zorica Janjetović, Anastasija Stokanović, Nataša Stojaković, Momir Mikov

**Affiliations:** ^1^Department of Family Medicine, Faculty of Medicine, University of Banja Luka, Banja Luka, Bosnia and Herzegovina; ^2^Primary Health Care Centre, Banja Luka, Bosnia and Herzegovina; ^3^Department of Pharmacology, Toxicology and Clinical Pharmacology, Faculty of Medicine, University of Banja Luka, Banja Luka, Bosnia and Herzegovina; ^4^Centre for Biomedical Research, Faculty of Medicine, University of Banja Luka, Banja Luka, Bosnia and Herzegovina; ^5^Department of Medical Biochemistry and Chemistry, Faculty of Medicine, University of Banja Luka, Banja Luka, Bosnia and Herzegovina; ^6^Department of Pharmacy, Faculty of Medicine, University of Banja Luka, Banja Luka, Bosnia and Herzegovina; ^7^Department of Hygiene, Faculty of Medicine, University of Banja Luka, Banja Luka, Bosnia and Herzegovina; ^8^Institute of Medical Statistics and Informatics, Faculty of Medicine, University of Belgrade, Belgrade, Serbia; ^9^Department of Dermatology, University of Alabama at Birmingham, Birmingham, AL, USA

## Abstract

**Background:**

Oxidative stress and inflammation are closely related pathophysiological processes, both occurring in type 2 diabetes mellitus (T2DM). In addition to the standard treatment of T2DM, a potential strategy has been focused on the use of bile acids (BAs) as an additional treatment. Ursodeoxycholic acid (UDCA), as the first BA used in humans, improves glucose and lipid metabolism and attenuates oxidative stress. The aim of this study was to evaluate the potential metabolic, anti-inflammatory, and antioxidative effects of UDCA in patients with T2DM.

**Methods:**

This prospective, double-blind, placebo-controlled clinical study included 60 patients with T2DM, randomly allocated to receive UDCA or placebo. Subjects were treated with 500 mg tablets of UDCA or placebo administered three times per day (total dose of 1500 mg/day) for eight weeks. Two study visits, at the beginning (F0) and at the end (F1) of the study, included the interview, anthropometric and clinical measurements, and biochemical analyses.

**Results:**

UDCA treatment showed a significant reduction in body mass index (*p* = 0.024) and in diastolic blood pressure (*p* = 0.033), compared to placebo. In addition, there was a statistically significant difference in waist circumference in the UDCA group before and after treatment (*p* < 0.05). Although no statistical significance was observed at the two-month follow-up assessment, an average decrease in glucose levels in the UDCA group was observed. After two months of the intervention period, a significant decrease in the activity of liver enzymes was noticed. Furthermore, a significant reduction in prooxidative parameters (TBARS, NO_2_^−^, H_2_O_2_) and significant elevation in antioxidative parameters such as SOD and GSH were found (*p* < 0.001).

**Conclusions:**

The eight-week UDCA administration showed beneficial effects on metabolic and oxidative stress parameters in patients with T2DM. Thus, UDCA could attenuate the progression and complications of diabetes and should be considered as an adjuvant to other diabetes treatment modalities. This trial is registered with NCT05416580.

## 1. Introduction

Diabetes mellitus (DM) is a common chronic disease causing life-threatening, disabling, and costly complications and reducing life expectancy. Worldwide in 2021, 537 million (10.5%) adults aged 20 to 79 already had diabetes, according to the data from the International Diabetes Federation (IDF). It is estimated that by 2045, 783 million (12.2%) adults will live with diabetes [1]. Among the ten countries with the highest prevalence of diabetes in the European region in 2015, Bosnia and Herzegovina was ranked fourth (9.9%), after Serbia in third (10.3%) while Turkey and Albania were in first and second place, respectively [2, 3].

Type 2 diabetes mellitus (T2DM) is the most prevalent form, contributing to 90-95% of all cases of diabetes [4]. It is a metabolic disorder manifested by hyperglycemia and insulin resistance (IR); allegedly, oxidative stress is the primary cause of this disorder [5]. T2DM is associated with multiple cardiovascular disease (CVD) risk factors, such as dyslipidemia, hypertension, inflammation, obesity, and oxidative stress. Oxidative stress is defined as an imbalance between the production and elimination of reactive oxygen species (ROS) in favor of their increased production [6, 7]. Mitochondria and endoplasmic reticulum are the main sources of ROS [8–10].

Oxidative stress and inflammation are closely related pathophysiological processes; one can be easily induced by the other and both are found in many pathological conditions [11] including T2DM. The current focus is on specific mechanism-based strategies that can target both oxidative stress and inflammatory pathways to improve the outcome of the disease burden [12].

A potential strategy in the therapy of T2DM is the use of bile acids (BAs), especially as an adjuvant therapy to other diabetes treatment modalities. Data from preclinical and clinical trials have shown that BAs, by activating the Takeda G-protein receptor 5 (TGR5) on enteroendocrine L cells, increase both the glucagon-like peptide-1(GLP-1) and insulin secretion, and postprandial glycemia, which might have a beneficial effect in T2DM [13–16]. A growing body of evidence supports the role of BA as mediators of the metabolic beneficial effects of therapies in the treatment of metabolic disorders [17].

Ursodeoxycholic acid (UDCA) is a hydrophilic BA that was discovered in bear bile, while in humans, it is only found as a secondary BA (1%-3% of total endogenous human BA production). UDCA is used as a medicine in the prevention of chronic hepatitis or cholestasis and in treatment of the primary biliary cholangitis [18, 19]. Studies show that UDCA improves the metabolism of glucose [20–22], alters the composition of BAs [23], attenuates oxidative stress or immune response, and exhibits anti-inflammatory properties that are especially important in metabolic diseases related to obesity [24–27]. UDCA is an important regulator of lipid metabolism; it also improves liver and adipose tissue mitochondrial function. Treatment of obese mice with UDCA resulted in reduced body weight and improved glucose metabolism. Such a drastic effect may be a consequence not only of the conversion of white to brown adipose tissue but also of the effects on other important metabolic organs, including the liver, which directly regulates lipid metabolism and nutrient absorption [28].

In accordance with previous observations, the aim of this study was to evaluate the potential metabolic, anti-inflammatory, and antioxidative effects of UDCA in patients with T2DM who are being treated with metformin but failed to achieve the target level of glycosylated hemoglobin (HbA1c) <7%. In these patients, the effects of UDCA on metabolic parameters and oxidative stress in T2DM patients were assessed.

## 2. Patients and Methods

### 2.1. Study Design and Study Population

**Figure 1:**
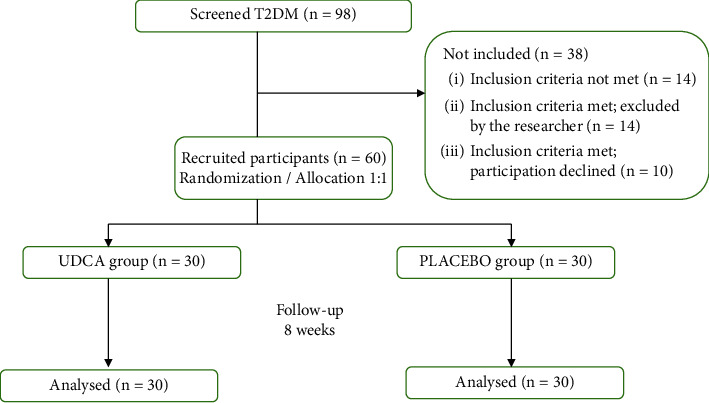
The study flowchart.

This was a prospective, double-blind, placebo-controlled clinical study with T2DM patients. It included 60 participants, 30 on UDCA and 30 on placebo treatment. The study was conducted at the Department of Family Medicine, Primary Health Care Centre (PHCC) in the city of Banja Luka, The Republic of Srpska, Bosnia and Herzegovina. Using the database of family medicine teams, eligible patients with T2DM were invited to participate in the study. The study protocol with detailed information was presented to 98 selected patients, and 60 of them who met the inclusion criteria for the study had to sign the informed consent prior to the study enrolment (Figure 1). The inclusion criteria were as follows: age 40 to 65 years, T2DM verified at least one year prior to the study enrolment, patients with an incomplete biochemical response and the value of HbA1c ≥ 6.5%, monotherapy (on metformin, maximally tolerated dose, up to 2000 mg/day), and body mass index (BMI) corresponding to overweight or obese (≥25 kg/m^2^). The exclusion criteria were as follows: patients with insulin or other injection treatment of T2DM, systemic administration of glucocorticoids (i.e., 10 days continuously) within 12 weeks prior to the study enrolment or other prior and concomitant immunosuppressant therapy, a medical history of cholecystitis, psychiatric disorders, acute and chronic kidney or liver disease, history of hypersensitivity to UDCA, malignancies, current pregnancy, and compliance issues.

### 2.2. Intervention

The study subjects were randomized 1 : 1 to 8 weeks of treatment with 500 mg tablets (2 tablets of 250 mg) of UDCA or a visually identical placebo administrated three times per day (total dose of 1500 mg/day). The study drug was obtained from the pharmaceutical and chemical industry “Bosnalijek” (Sarajevo, Bosnia and Herzegovina). Randomization was performed using a unique scheme unknown to the investigator with 30 patients in the treatment group and 30 in the placebo group. The participants, as well as the investigators, were blinded to the study drug allocation. During the follow-up, patients were asked to register in the treatment log diary any reaction or possible adverse effects. Each report was registered, and causality was analyzed. To ensure compliance for all study participants, a weekly follow-up through telephone conversation was performed.

### 2.3. Anthropometric and Clinical Measurements

Study visits were carried out at the beginning (F0) and at the end of the study (F1). At F0 and F1 visits, the interview (UDCA T2DM questionnaire) and physical examination (anthropometric and clinical measurements) were performed. The sociodemographic and clinical data such as age, gender, education and occupation, duration of T2DM, related comorbidities, and current medication were collected. Anthropometric and clinical measurements included body weight and height, waist circumference (WC), body mass index (BMI), and blood pressure—systolic blood pressure (SBP) and diastolic blood pressure (DBP). Anthropometric measurements were performed in accordance with the Clinical Guideline for Primary Health Care [29]. BMI was calculated as weight (kg) divided by the square of height (m^2^) according to the WHO [30]. Blood pressure was assessed using a mercury sphygmomanometer GIMA, CE 0476, Italia. The cusp was placed around the left upper arm 2.5 cm above the cubital fossa. A participant was in a sitting position resting for 10 minutes prior to blood pressure measurement; three measurements were obtained five minutes apart. The mean value expressed in millimeters of mercury (mmHg) was used for the data collection in accordance with the American Heart Association recommendations [31].

### 2.4. Biochemical Analyses

Blood samples were taken at the beginning of the study (F0) and following the intervention period (F1). Serum levels of fasting glucose, lipid profile: triglycerides (TG), total cholesterol (TC), high-density lipoprotein cholesterol (HDL-C), low-density lipoprotein cholesterol (LDL-C), and, from whole blood, glycosylated hemoglobin (HbA1c) were measured utilizing the enzymatic methods on the biochemical analyzer (Abbott Alinity c). Liver enzymes including aspartate aminotransferase (AST), alanine aminotransferase (ALT), gamma-glutamyl transferase (GGT), and alkaline phosphatase (ALP) were measured to assess liver function, and the IFCC method of reference (with pyridoxal phosphate) was used. LDL-C was calculated by the Friedewald formula: LDL − C (mg/dL) = TC − (HDL − C + TG/5). Others, such as fasting insulin and proinflammatory markers: C reactive protein (CRP) and interleukin-6 (IL-6), were determined using the automatic immunoassay analyzer (ECLIA Cobas PRO). Reference values for IL-6 were 0-7.7 pg/mL. Insulin resistance was defined as the value of the insulin resistance index, HOMA IR ≥ 2.5; it was determined using the Homeostatic Model Assessment (HOMA IR) index, calculated as the product of fasting insulin (mIU/L) and fasting glucose levels (mmol/L) divided by 22.5 [32].

### 2.5. Oxidative Stress Parameters

We exploited plasma and erythrocyte lysate to establish the values of oxidative stress parameters. The following methods are used: nitro blue tetrazolium (NTB) reduction [33] for measuring superoxide anion radical (O_2_^−^), Pick and Keisari [34] for measuring hydrogen peroxide (H_2_O_2_), a green method [35] for measuring nitrite (NO_2_^−^), TBA [36] for measuring the index of lipid peroxidation and thiobarbituric acid reactive substances (TBARS), and Beutler's methods [37–41] for measuring catalase (CAT), superoxide dismutase (SOD), and reduced glutathione (GSH).

### 2.6. Statistical Analyses

Results are presented as count (%), means ± standard deviation, or median (interquartile range) depending on data type and distribution. Groups are compared using parametric (*t*-test) and nonparametric (chi-square, Mann–Whitney *U* test, and Wilcoxon's signed-rank test) tests. All *p* values less than 0.05 were considered significant. All data were analyzed using SPSS 29.0 (IBM Corp. Released 2023. IBM SPSS Statistics for Windows, Version 20.0. Armonk, NY: IBM Corp.) or R 3.4.2. (R Core Team (2017, Foundation for Statistical Computing, Vienna, Austria. URL https://www.R-project.org/.)

### 2.7. Ethics Approval and Consent to Participate

The study was conducted in accordance with the Helsinki Declaration and approved by the Ethics Committee for Research in Humans and Biological Materials of the Faculty of Medicine, University of Banja Luka (no. 18/4.92/22). Informed consent was obtained from each participant involved in the study. This trial is registered at http://www.clinicaltrial.gov/ as NCT05416580.

## 3. Results

Sixty patients with T2DM were included in this clinical study. The average age was 56.3, and 33 participants were males (55%). The average age and gender distribution were similar in both groups, as well as the duration of diabetes mellitus (Table 1 part a). The demographic and clinical characteristics at baseline and upon study completion are succinctly summarized in Table 1 parts a and b. Notably, no significant differences were observed between the treatment group and placebo group with respect to all baseline characteristics.

The majority in both groups were in cohabitation (86.7% in UDCA, 73.3% in placebo; *p* = 0.197) and employed (66.7% in UDCA, 56.7% in placebo; *p* = 0.426). The CVD was observed in 63.3% of patients in the UDCA group and 76.7% of patients in the placebo group. Notably, 66.7% of patients were receiving antihypertensive medications within the UDCA group, as compared to 73.3% of patients in the placebo group. Additionally, approximately one-quarter of the participants in both groups were using statins, with no statistically significant differences between the two groups.

The baseline anthropometric values (BMI and WC) were very similar in both groups; they were even several units higher in the UDCA group. Regarding the BMI, while no significance was observed at the baseline, a significantly higher average decrease was observed in the UDCA group after 8 weeks of treatment, compared to placebo (−0.5 ± 0.7 vs. −0.2 ± 0.5; *p* = 0.024). Also, a significant decrease in the intragroup change compared to baseline values in UDCA was found (31.9 ± 5.7 vs. 32.4 ± 5.9; *p* < 0.05). A significant decrease in WC in intragroup change compared to baseline values in UDCA was observed (102 ± 11 vs. 106.7 ± 11.2; *p* < 0.05).

Besides anthropometry, both blood pressures, SBP and DBP, were analyzed. At the beginning of the trial, there were no differences between the groups in BP. The significant difference in average decrease in DBP (Table 1 part b) was observed in the UDCA group, compared to the placebo group (−1.5 ± 4.9 vs. 2 ± 7.3; *p* = 0.033).

The values of glucoregulation parameters (glucose, insulin, HOMA IR, and HbA1c) and lipids (total cholesterol, triglycerides, HDL-C, and LDL-C) are presented in Table 2. The average glucose and HbA1c levels were very similar in both groups at the beginning of the trial, with no significant differences. However, the comparative analysis of postintervention measures between the study groups failed to yield a statistically significant difference regarding glucoregulation parameters, insulin resistance, and lipid profile. While no statistical significance was observed at the two-month follow-up assessment, the average decrease in glucose levels was revealed within the UDCA treatment group. It is noteworthy that no significant differences were revealed in the lipid status in the UDCA group after eight weeks of treatment.

Table 3 presents the baseline and end-of-study results for values of liver enzymes (AST, ALT, ALP, and GGT) and inflammatory parameters (CRP and IL-6) in both the UDCA and placebo groups. There were no significant differences in liver enzyme and inflammation parameter levels between the groups at baseline. However, at the end of the study, both groups exhibited a significant reduction in *Δ*ALT, as well as in *Δ*GGT, with median (IQR) values of -6 (13) vs. -2.5 (12) (*p* = 0.01) and *Δ*GGT -3.5 (16) vs. 2 (12) (*p* < 0.001), respectively, in the within-group analysis. Also, a significant decrease in the intragroup change of AST (22 (7) vs. 26 (8); *p* < 0.05), ALT (27.5 (14) vs. 38.5 (23); *p* < 0.05), and GGT (24 (19) vs. 27.5 (31); *p* < 0.05) compared to baseline values in the UDCA group was found.

Inflammatory parameters did not change significantly. A significant decrease in the intragroup change was found in CRP (1.4 (2.3) vs. 1.9 (2.4); *p* < 0.05) compared to baseline values in the UDCA group.

**Figure 2:**
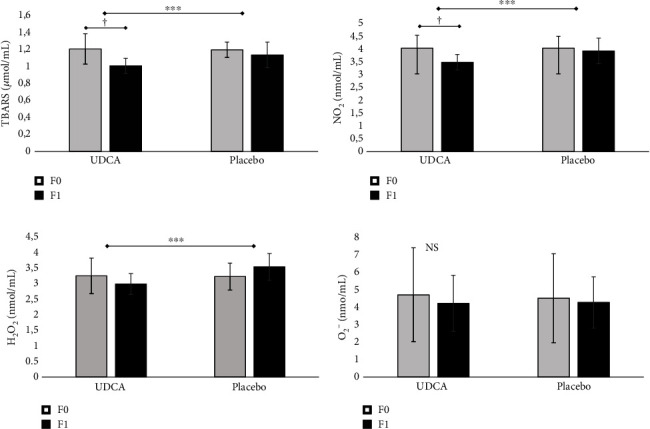
The effects of eight weeks of UDCA/placebo treatment on prooxidative stress parameters in T2DM patients (a–d). The thiobarbituric acid reactive substances (TBARS), NO_2_, H_2_O_2_, and O_2_^−^ values at the beginning (F0) and at the end of the study (F1). Independent sample *t*-test was done, and asterisk (^∗^) indicates significant differences between the groups, ^∗∗∗^*p* < 0.001. Paired sample *t*-test was used to test for changes within the group, and the dagger sign (†) indicates significant differences within the groups, †*p* < 0.05.

**Figure 3:**
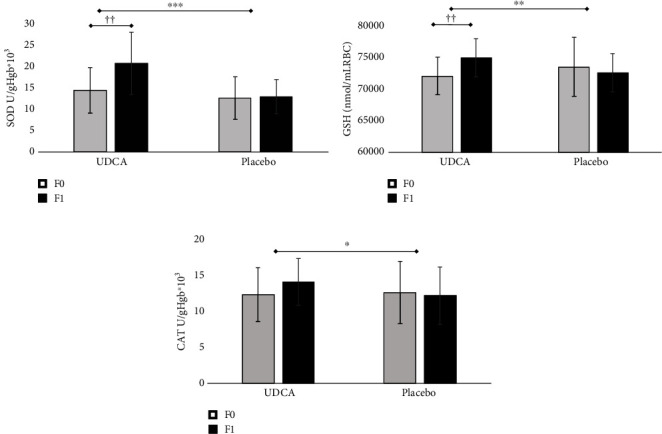
The effects of eight weeks of UDCA/placebo treatment on antioxidative stress parameters in T2DM patients (a–c). The superoxide dismutase (SOD), catalase (CAT), and glutathione (GSH) at the beginning (F0) and at the end of the study (F1). Independent sample *t*-test was done, and asterisk (^∗^) indicates significant differences between the groups, ^∗^*p* < 0.05, ^∗∗^*p* < 0.01, and ^∗∗∗^*p* < 0.001. Paired sample *t*-test was used to test for changes within the group, and the dagger sign (†) indicates significant differences within the groups, ††*p* < 0.01.

Finally, both prooxidative and antioxidative parameters within both study groups at the beginning and culmination of the intervention period were monitored (Figures 2 and 3). Initial observations revealed no discernible discrepancies in oxidative status between the groups. However, as the intervention period drew to a close, noteworthy and statistically significant alterations occurred within the UDCA-treated group. Specifically, the group receiving UDCA exhibited a statistically significant reduction in prooxidative parameters (TBARS, NO_2_^−^, and H_2_O_2_) and a concurrent, statistically significant elevation in antioxidative parameters (SOD and GSH). Furthermore, significant intragroup changes were also reported (*p* < 0.05) after the follow-up period. CAT revealed significantly higher values on follow-up between groups (14.1 ± 3.2 vs. 12.2 ± 4; *p* = 0.048), but no significant change was observed (Figure 3).

## 4. Discussion

In this study, UDCA was used for the cotreatment of patients with T2DM. Although the medicinal effects of BAs were known for many years, a great interest in these drugs appeared at the end of the last century. However, most experimental studies with these drugs have been conducted in animals (rodents). Some clinical studies have demonstrated the beneficial effects of BAs, particularly UDCA, in liver diseases, and other gastrointestinal pathology [42–44]. To unravel the underlying mechanism of UDCA on diabetes mellitus in humans, a clinical trial in patients with T2DM was conducted. A daily oral dose of 1500 mg of UDCA was administered to the study subjects for eight weeks. As a previous study showed [45], the short-term changes in HbA1c are observed eight weeks after the medication alteration. During this period, the effects of this drug on diabetes, specifically metabolic, anti-inflammatory, and antioxidative outcomes, were monitored. T2DM is usually accompanied by other modifiable CVD risk factors, such as hypertension, dyslipidemia, and obesity. Therefore, it was important to monitor these factors in diabetic patients as well.

The results of the present study showed a significant reduction in BMI in the UDCA group after eight weeks of treatment, compared to placebo. This finding is of great importance since weight loss is a part of lifestyle management of T2DM patients. In accordance with that notion, the study in animals revealed that UDCA led to a reduction of lipogenesis and gluconeogenesis in obese mice, suggesting that UDCA plays an important role in lipid and glucose metabolism and energy storage capacity in connection with obesity. UDCA is equally effective in reducing whole-body adiposity [28].

Elevated arterial BP contributes to increased incidence of both micro- and macrovascular complications in patients with T2DM. Namely, the coexistence of these two major risk factors leads to a fourfold increased risk for cardiovascular disease (CVD) as compared to normotensive nondiabetic individuals [46]. In this study, UDCA exhibited a positive effect on BP, particularly on DBP. Eight-week-treatment with UDCA significantly reduced DBP in T2DM patients, compared to placebo. In accordance with strict inclusion criteria and randomization of the present study, no differences between the examined groups in the age and gender of study participants, as well as the duration of diabetes were found. As an incurable disease, strict metabolic control in T2DM (glucoregulation, lipids, and blood pressure) is a very important element of the treatment, as well as in the prevention of diabetic complications [47, 48]. Glycemic control is the central focus of managing diabetes and its complications. Early reports indicate that UDCA improves glucose metabolism; that is, administration of high-dose UDCA improves glycemic parameters, insulin sensitivity, and insulin resistance surrogate markers in patients with nonalcoholic steatohepatitis [20, 21]. A meta-analysis, including 17 studies focusing only on glucoregulation in 2,950 patients with T2DM found a 0.55% improvement in HbA1c and fasting glycemia after using BA sequestrants [49]. Our data revealed a visible, but not statistically significant, decrease in fasting blood glucose and HbA1c levels in the UDCA treatment group. Other glucose parameters, such as insulin and HOMA IR, did not change significantly either.

A meta-analysis of 15 different studies in humans, including patients with DMT2 and the effects of BAs and sequestrants, showed that lipid levels (LDL-C, HDL-C, and triglycerides), as well as HbA1c, improved upon BA treatment [50]. A recent experimental study in mice with diabetes divulged the biological effects of the treatment with the antilipemic probucol together with UDCA (microcapsules) drugs, resulting in hypoglycemic and anti-inflammatory effects in these animals [51]. UDCA, used as a part of the combination therapy, supported the favorable therapeutic effects of the basic drug and its bioavailability. Additionally, other studies utilized the nano- and microparticle approach for better oral drug delivery in combination with BAs [52–55]. Nevertheless, the results of this study revealed that lipid status remained unaffected, along with the inflammatory parameters (IL-6 and CRP). The possible explanation for this effect could stem from the oral bioavailability of the drug that might not be sufficient to hinder inflammation unless combined with some other drugs.

The hepatoprotective effects of UDCA are already known. UDCA is a well-established drug approved for the treatment of primary biliary cholangitis and primary sclerosing cholangitis, with an adequate safety profile and minimal side effects, even at high doses [42, 43]. Alonso-Peña et al. [56] found that treatment with UDCA normalized aminotransferase levels and that the drug can also efficiently attenuate liver damage in patients with unexplained hypertransaminasemia. The study conducted by Mueller et al. [57] reported that the three-week-long administration of UDCA in obese patients, at a dose of 20 mg/kg/day, reduced the serum AST, GGT, free fatty acids, total cholesterol, and LDL-C. Robles-Díaz et al. [58] emphasized the role of UDCA in the treatment and prevention of drug-induced liver injury. In addition, the relatively common empiric use of UDCA in these conditions appears to be safe and led to reductions in bilirubin and transaminase levels.

In accordance with the described effects of UDCA on liver enzyme profile, we have found a significant reduction in liver enzymes, ALT, and GGT in the UDCA group compared to placebo. Despite the high doses of UDCA, minimal side effects and good compliance were observed. UDCA achieves its cytoprotective and antiapoptotic effects by preventing the formation of ROS [59, 60]. However, measurement of ROS is still difficult and one of many available methods is lipid peroxidation and TBARS, as useful markers of disease risk and progression [61]. Hyperglycemia is associated with the massive production of ROS. In the present study, the antioxidative effects of UDCA, as measured through monitoring levels of ROS and the activity of GSH, SOD, and CAT, were observed. The results of this study demonstrated a significant reduction of all prooxidative parameters: TBARS, NO_2_^−^, and H_2_O_2_, during the 8-week-long period of UDCA treatment. However, the level of O_2_^−^ remained unaffected. During the follow-up, it was also found that the level of antioxidative molecules such as SOD and GSH significantly increased by UDCA treatment. At the same time, the CAT level was also increased, but that increase was not significant.

This study revealed the beneficial effects of UDCA in T2DM patients as an auxiliary treatment to metformin. In addition to a small number of clinical studies performed on patients, the strength of this study is its contribution to the treatment of T2DM. Besides, the design of this study, as a double-blind randomized clinical trial with parallel groups and strong inclusion/exclusion criteria, makes the obtained results outstanding. This study is a small-scale study with a limited number of participants during the COVID-19 pandemic performed in one center, thus creating a major limitation to this study. The results of this study are promising, and more research is warranted to explore all the benefits of UDCA in T2DM.

## 5. Conclusions

The study demonstrated that an eight-week UDCA administration had beneficial effects on anthropometric status, liver function, and diastolic blood pressure in patients with T2DM. UDCA significantly improved BMI, diastolic blood pressure, liver enzymes (ALT and GGT), and oxidative stress parameters, thus potentially attenuating the progression and complications of diabetes.

## Figures and Tables

**(a):** 

Parameters	UDCA	Placebo	*p* value
Age (yrs.)	56.4 ± 6.6	56.1 ± 6.3	0.842^a^
Gender male	17 (56.7%)	16 (53.3%)	0.795^c^
DM duration (years)			
<5	11 (36.7%)	15 (50%)	0.181^b^
5-9.9	12 (40%)	12 (40%)	
10-14.9	5 (16.7%)	2 (6.7%)	
15+	2 (6.7%)	1 (3.3%)	

All values are expressed as mean ± SD or *n* (%). ^a^Independent sample *t*-test. ^b^Mann–Whitney *U* test. ^c^Pearson's chi-square test. DM: diabetes mellitus.

**(b):** 

Parameters	UDCA (*n* = 30)			Placebo (*n* = 30)		
	Baseline (F0)	8 weeks (F1)	8 weeks—baseline (*Δ*)	Baseline (F0)	8 weeks (F1)	8 weeks—baseline (*Δ*)
Anthropometry and BP						
BMI (kg/m^2^)	32.4 ± 5.9	31.9 ± 5.7	−0.5 ± 0.7†	31.2 ± 5.3	31.1 ± 5.4	−0.2 ± 0.5^∗^
WC (cm)	106.7 ± 11.2	102 ± 11	−4.7 ± 4.5†	101 ± 13.3	98.5 ± 11.7	−2.5 ± 4.4†
BP systolic (mmHg)	134.8 ± 17.4	131.7 ± 14.2	−3.2 ± 14.6	130 ± 16.1	128.7 ± 19.3	−1.3 ± 16.2
BP diastolic (mmHg)	83.8 ± 7.4	82.3 ± 6.9	−1.5 ± 4.9	82.2 ± 8.1	84.2 ± 8.6	2 ± 7.3^∗^

All values are expressed as mean ± SD. ^∗^*p* < 0.05: Independent sample *t*-test compared with UDCA group. †*p* < 0.05: Wilcoxon's signed ranks test compared 8 weeks and baseline in the UDCA group or compared 8 weeks and baseline in the placebo group. F0: beginning of study; F1: end of study; WC: waist circumference; BP: blood pressure.

**Table 2:** Glucoregulation and lipid status at baseline and after eight weeks of UDCA or placebo treatment.

Parameters	UDCA (*n* = 30)			Placebo (*n* = 30)		
	Baseline (F0)	8 weeks (F1)	8 weeks—baseline (*Δ*)	Baseline (F0)	8 weeks (F1)	8 weeks—baseline (*Δ*)
Glucoregulation						
Glucose (mmol/L)	9.1 ± 3.9	8.5 ± 2.7	−0.6 ± 2.5	8.8 ± 3.0	9.0 ± 3.0	0.2 ± 1.5
HbA1c (%)	7.2 ± 1.8	7.0 ± 1.5	−0.2 ± 0.6	7.3 ± 1.6	7.2 ± 1.6	0 ± 0.6
Insulin (mIU)	11.2 (6.5)	11.1 (7.3)	-0.9 (5.2)	10.7 (5.1)	10.4 (5)	-0.3 (4.2)
HOMA IR	3.9 (4.4)	4.0 (2.9)	-0.4 (2)	4.3 (4)	4.4 (3.5)	-0.2 (1.3)
Lipids						
Cholesterol (mmol/L)	5.8 ± 1.4	5.3 ± 1.2	−0.5 ± 1.5	5.1 ± 1.3	5.1 ± 1.1	0.0 ± 1.0
Triglycerides (mmol/L)	2.1 (2)	1.9 (1.1)	-0.1 (1.2)	1.8 (1.6)	2.2 (1.7)	0.1 (0.9)
HDL-C (mmol/L)	1.2 ± 0.2	1.1 ± 0.2	−0.1 ± 0.1	1.1 ± 0.2	1.1 ± 0.3	−0.1 ± 0.2
LDL-C (mmol/L)	3.4 ± 1.2	3.2 ± 0.9	−0.2 ± 1.3	2.8 ± 1.3	3.0 ± 1.0	0.2 ± 1

All values are expressed as mean ± SD or median (IQR). F0: beginning of study; F1: end of study; HbA1c: glycosylated hemoglobin A1c; HOMA IR: Homeostatic Model Assessment index; HDL-C: high-density lipoprotein cholesterol; LDL-C: low-density lipoprotein cholesterol.

**Table 3:** The change of liver enzymes and inflammatory parameters at baseline and after eight weeks of UDCA or placebo treatment.

Parameters	UDCA (*n* = 30)			Placebo (*n* = 30)		
	Baseline (F0)	8 weeks (F1)	8 weeks—baseline (*Δ*)	Baseline (F0)	8 weeks (F1)	8 weeks—baseline (*Δ*)
Liver enzymes						
AST (UI)	26 (8)	22 (7)	-3.5 (6)†	23.5 (9)	21.5 (11)	0 (8)
ALT (UI)	38.5 (23)	27.5 (14)	-6 (13)†	31 (18)	26.5 (14)	-2.5 (12)^∗^
ALP (UI)	51 (24)	55 (23)	5 (14)	62 (15)^∗^	61 (21)	2.5 (22)
GGT (UI)	27.5 (31)	24 (19)	-3.5 (16)†	28.5 (18)	27 (21)^∗^	2 (12)^∗∗∗^
Inflammatory						
CRP(mg/L)	1.9 (2.4)	1.4 (2.3)	-0.4 (0.7)†	2.5 (2.7)	2.6 (2.5)	-0.2 (1.2)
IL-6 (pg/mL)	2.4 (2.1)	2.1 (1.9)	0 (1.8)	3.3 (4.3)^∗^	2.7 (1.8)	-0.5 (2)

Data are expressed as median (IQR). ^∗^*p* < 0.05, and ^∗∗∗^*p* < 0.001: Mann–Whitney *U* test compared with UDCA group. †*p* < 0.05: Wilcoxon's signed rank test compared 8 weeks and baseline in the UDCA group. F0: beginning of study; F1: end of study; AST: aspartate aminotransferase; ALT: alanine aminotransferase; ALP: alkaline phosphatase; GGT: gamma-glutamyl transferase; CRP: C reactive protein; IL-6: interleukin-6.

## Data Availability

Supporting data is not applicable.
